# Dissection of a sensorimotor circuit underlying pathogen aversion in *C. elegans*

**DOI:** 10.1186/s12915-022-01424-x

**Published:** 2022-10-08

**Authors:** Adam Filipowicz, Jonathan Lalsiamthara, Alejandro Aballay

**Affiliations:** 1 Department of Molecular Microbiology & Immunology, Oregon Health & Science University, Portland, OR 97239 USA; 2 Neuroscience Graduate Program, Oregon Health & Science University, Portland, OR 97239 USA

**Keywords:** Behavioral immunity, *Caenorhabditis elegans*, Neural circuits, Pathogen avoidance, Whole-brain modeling

## Abstract

**Background:**

Altering animal behavior to reduce pathogen exposure is a key line of defense against pathogen attack. In *Caenorhabditis elegans*, alterations in intestinal physiology caused by pathogen colonization and sensation of microbial metabolites may lead to activation of pathogen aversive behaviors ranging from aversive reflexes to learned avoidance. However, the neural circuitry between chemosensory neurons that sense pathogenic bacterial cues and the motor neurons responsible for avoidance-associated locomotion remains unknown.

**Results:**

Using *C. elegans*, we found that backward locomotion was a component of learned pathogen avoidance, as animals pre-exposed to *Pseudomonas aeruginosa* or *Enterococcus faecalis* showed reflexive aversion to drops of the bacteria driven by chemosensory neurons, including the olfactory AWB neurons. This response also involved intestinal distention and, for *E. faecalis*, required expression of TRPM channels in the intestine and excretory system. Additionally, we uncovered a circuit composed of olfactory neurons, interneurons, and motor neurons that controls the backward locomotion crucial for learned reflexive aversion to pathogenic bacteria, learned avoidance, and the repulsive odor 2-nonanone.

**Conclusions:**

Using whole-brain simulation and functional assays, we uncovered a novel sensorimotor circuit governing learned reflexive aversion. The discovery of a complete sensorimotor circuit for reflexive aversion demonstrates the utility of using the *C. elegans* connectome and computational modeling in uncovering new neuronal regulators of behavior.

**Supplementary Information:**

The online version contains supplementary material available at 10.1186/s12915-022-01424-x.

## Background

To reduce exposure to pathogens, organisms throughout the animal kingdom, including humans, engage in behavioral immune activities [1–4]. Mechanistic insights into behavioral immunity are therefore crucial in furthering our understanding of human health and disease. Numerous studies have shown that *Caenorhabditis elegans* exhibits behavioral immunity in the form of pathogen avoidance strategies that improve its survival [4–9]. These behaviors range from aversive reflexes to learned avoidance. Studies using pathogenic bacteria such as *Pseudomonas aeruginosa* and *Enterococcus faecalis*, for example, have shown that these bacteria are initially attractive to *C. elegans*, and it is only after hour-long exposure to the pathogens that the valence of the bacteria switches from attractive to aversive [9, 10]. This is in contrast to the *C. elegans* avoidance of the toxins produced by *Streptomyces*, which takes place in a matter of seconds [7].

Taking advantage of the genetic tractability of *C. elegans*, the neuronal and molecular mechanisms of these behaviors have begun to be made clear. For example, the response to *Streptomyces* requires the G-protein-coupled receptor (GPCR) SRB-6, which is expressed in five different chemosensory neurons: ASH, ADL, ADF, PHA, and PHB [7]. ASH, in particular, was shown to respond to both *Streptomyces* in an *srb-6-*dependent manner, though whether loss of ASH function leads to an abrogated behavioral response remains unknown. The avoidance of *P. aeruginosa* and *E. faecalis*, on the other hand, seems to involve a variety of bacterial cues sensed by various chemosensory neurons. These include bacterial metabolite sensation by DAF-7-expressing ASI and ASJ chemosensory neurons [11, 12], olfactory preference establishment by AWB and AWC olfactory neurons and modulation by serotonergic ADF neurons and RIA interneurons [9, 13], oxygen sensation by NPR-1 expressing AQR, PQR, and URX neurons [8, 14, 15], nitric oxide sensation by ASJ neurons [16], and BAG neuron-mediated sensation of carbon dioxide [17].

Signals from the sensory neurons described above likely converge on downstream interneurons. Activation of ASI neurons, for example, results in the release of the insulin-like-peptide INS-6, inhibiting *ins-7* expression in URX and subsequent upregulation of DAF-2 activity in RIA interneurons [18]. These same interneurons are involved in the modulation of olfactory preference [13] and thus may be important integrators of distinct bacterial cues triggering avoidance behaviors.

Any chemosensory signal must eventually be propagated down not only to interneurons but also to the motor neurons required for avoidance-associated locomotion [19–21]. The circuitry linking these neurons together is unknown, but the complete connectome of *C. elegans* [22] can be used to dissect the mechanisms involved in translating the detection of pathogenic cues into physical avoidance. We discovered that backward locomotion is a crucial component of pathogen avoidance, as animals trained on either *P. aeruginosa* or *E. faecalis* display reflexive aversion to these pathogens. To elucidate the reflexive aversion circuitry, we used simulations of the *C. elegans* nervous system, which have been shown to be useful in studying behaviorally relevant neural activity [23, 24]. Using one such simulation platform, the *C. elegans* Neural Interactome [24], we investigated the neural patterns resulting from stimulation of the chemosensory neurons known to be involved in different pathogen avoidance behaviors. We found that oscillations in motor neurons critical for backward locomotion could be induced by stimulation of the olfactory AWB neurons. AUA and RMG interneurons electrically coupled to AWB neurons also showed high activity upon AWB stimulation, and in silico ablation of these neurons resulted in the loss of motor neuron oscillations. Genetic ablation of these neurons demonstrated their involvement not only in pathogen avoidance but 2-nonanone aversion as well. The olfactory neuron AWB electrically synapses onto AUA and RMG interneurons, which themselves synapse onto motor command interneurons to control backward locomotion motor neurons, thus forming a novel sensorimotor circuit for pathogen and repulsive odor aversion. These findings mark the first identification of a complete sensorimotor circuit for pathogen avoidance and suggest a way forward for future studies to integrate computational and experimental tools in the dissection of behaviorally relevant neural circuits.

## Results

### Intestinal infection by *P. aeruginosa* or *E. faecalis* induces a learned reflexive aversion requiring multiple chemosensory neurons

**Fig. 1.**
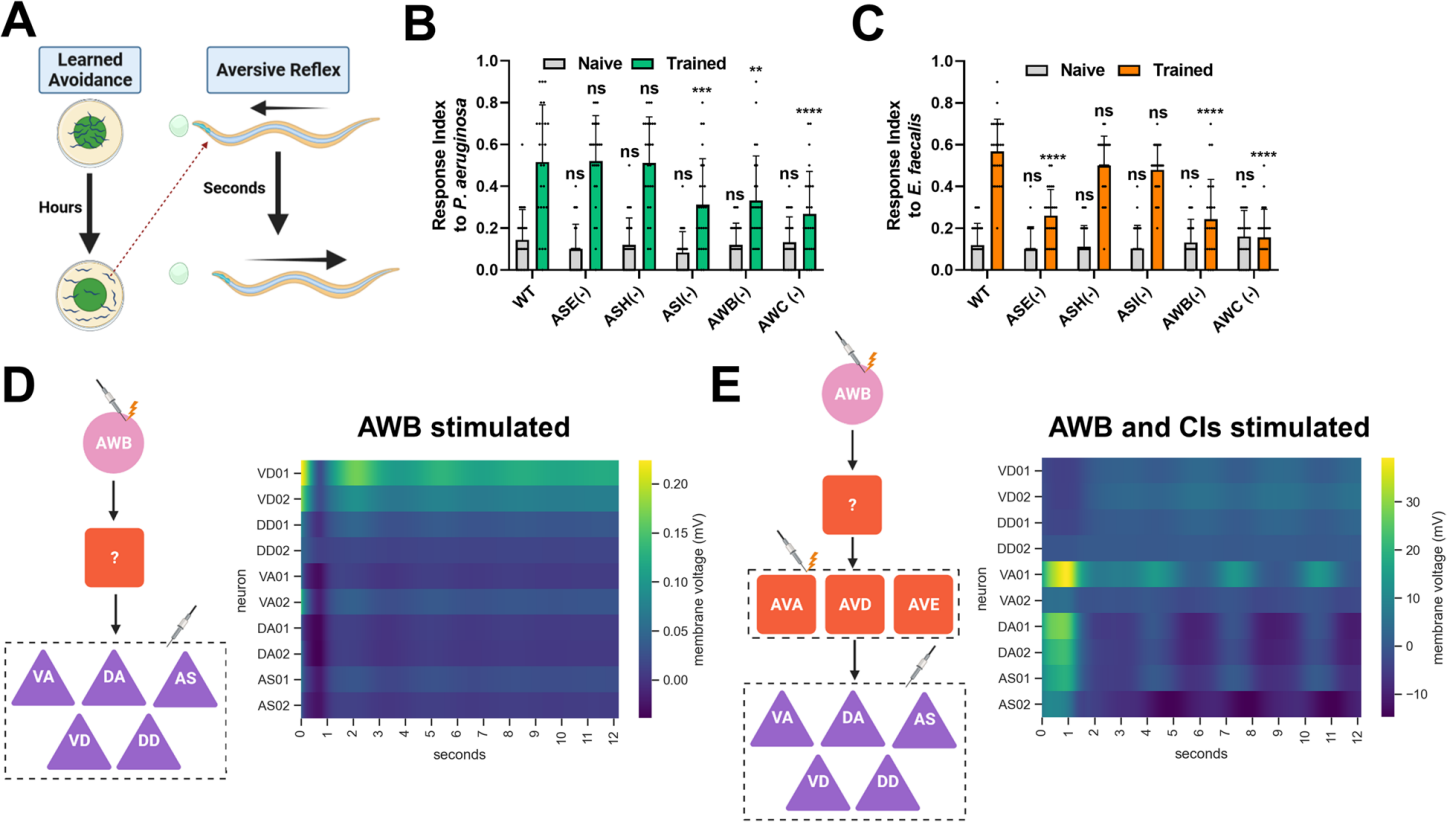
Intestinal infection induces a learned reflexive aversion requiring multiple chemosensory neurons. **A** Diagram of the assays to determine learned avoidance (left) and naïve reflexive aversion (right). Details of assays can be found in the Materials and Methods. **B** Response index to *P. aeruginosa* for both naïve (gray) and trained (green) animals with either no neurons ablated (N2, WT) or ASE (PR680), ASH (JN1713), ASI (PY7505), AWB (JN1715), or AWC (PY7502) neurons ablated. **C** Response index to *E. faecalis* for the same groups as in **B**. For both **B** and **C**, two-way ANOVA with subsequent comparison to naïve or trained WT groups was performed. Error bars depict standard deviation. *N* = 25 (individual dots) for all groups. **D** Schematic of the sensorimotor circuit and protocol used in the Neural Interactome (left). AWB neurons were stimulated at 5.0 nA, and the activity of VA, DA, AS, VD, and DD motor neurons (dashed-outline) was recorded. There is no direct connection between AWB neurons and the motor neurons, so an unknown interneuron must complete the circuit (question mark). Pink circle = sensory neuron; red square = interneuron; purple triangle = motor neuron. The recorded activity of the motor neurons is presented as a heatmap (right), with rows representing individual neuronal membrane voltage (in millivolts (mV)) over time (in seconds). The first two neurons of each motor neuron class were chosen for ease of visualization. **E** An updated schematic of the stimulation protocol (left). AVA, AVD, and AVE command interneurons (CIs) were stimulated at 0.9 nA along with the 5.0 nA stimulation of AWB neurons. This resulted in oscillations in the motor neurons (right). There is no direct connection between AWB neurons and the CIs, so another interneuron must complete the circuit (question mark)

To investigate the neural circuitry governing the translation of pathogen chemosensory cues into the motor neuron activity necessary for avoiding said cues, we directly tested avoidance locomotion. We made use of an assay that would allow us to quickly assess individual neuron requirements for reflexive aversion both before and after exposure to a pathogen (Fig. 1A). The pathogenic bacteria *P. aeruginosa* and *E. faecalis* are initially attractive to *C. elegans* and only induce an avoidance response after many hours of exposure [4, 9, 10, 13]. This learning process involves the association of infection and subsequent physiological responses, including intestinal distention, engagement of RNAi pathways, and immune activation, with bacterial cues, resulting in avoidance of the bacteria [10, 12, 25]. Using the reflexive aversion assay, we found that naïve animals do not respond to drops of *P. aeruginosa* (Fig. 1B) or *E. faecalis* (Fig. 1C). Unexpectedly, animals exposed to bacteria prior to testing showed reflexive aversion to the same bacteria. The response to *P. aeruginosa* required ASI, AWB, and AWC neurons, while the response to *E. faecalis* required ASE, AWB, and AWC neurons (Fig. 1B, C). These results indicate that intersecting neural circuits are required for learned reflexive aversion against different pathogens and represent the discovery of a novel behavioral response to *P. aeruginosa* and *E. faecalis*.

### Nervous system simulation predicts that AUA and RMG neurons are in the AWB neuron-mediated learned reflexive aversion circuit

To uncover the overall neural circuitry that integrates bacterial-related cues that result in learned reflexive aversion, we used the *C. elegans* Neural Interactome, a simple, user-friendly simulation of the *C. elegans* nervous system that allows for stimulation and ablation of individual neurons and outputs total network activity [24]. The simulation takes advantage of the complete connectome of *C. elegans* and its neural dynamics and was previously shown to assist in the study of neural response patterns associated with locomotion and external stimuli such as nose touch. This makes the Neural Interactome a potentially useful tool in studying other aversive behaviors, including pathogen avoidance. To assess the neural activity of reflexive aversion, we measured oscillations in VA, DA, VD, DD, and AS motor neurons as a readout for backward locomotion, as it has previously been shown that these neurons are active during backward locomotion and function as oscillators [19, 20, 24, 26]. Because AWB neurons are at the intersection of the circuits required for learned reflexive aversion against *P. aeruginosa* and *E. faecalis*, we first stimulated AWB neurons. However, we found that motor neurons showed very weak maximum amplitudes shortly after AWB stimulation (VA01, for example, had a maximum membrane voltage of .014 mV) with small oscillations that tapered off throughout the 12-second simulation (Fig. 1D and Additional file 1: Fig. S1A), indicating that additional neurons might participate in the circuit. Previous studies indicated that the command interneurons (CIs) AVA, AVD, and AVE are necessary for reflexive aversion to nose touch [21, 24]. Thus, we used the Neural Interactome to activate AWB together with CIs and found oscillatory activity in the VA, DA, VD, DD, and AS motor neurons (Fig. 1E and Additional file 1: Fig. S1B). Most motor neurons showed strong initial activity (for example, VA01 maximum membrane voltage was 39.2 mV), followed by a dip in voltage and then oscillating activity with periods of about 2.5 s. VA, DA, and AS motor neuron oscillations were in phase with one another, but out of phase with VD and DD motor neurons, which a previous report indicated are required to coordinate backward movement [24]. Stimulation of AWC and ASI neurons led to no oscillations in the motor neurons, while stimulation of ASE neurons led to fast, but very weak oscillations (Additional file 2: Fig. S2). This indicates that AWB neurons are the primary mediator of reflexive aversion, while AWC, ASI, and ASE play some other roles in the learning process.

To confirm that motor neuron oscillations are a good readout for aversion behaviors, we examined a behavior that has a known circuit correlate: reflexive aversion to the common laboratory detergent sodium dodecyl sulfate (SDS [27];). We confirmed that this behavior requires the chemosensory neuron ASH, as animals lacking ASH neurons responded less strongly to SDS compared to wild-type animals (Additional file 3: Fig. S3A). Aversion to SDS requires the command interneurons AVA, AVD, and AVE, and the motor neurons VA, DA, VD, DD, and AS [19, 20, 27]. The connections between these neurons resolve as a three-layer circuit (Additional file 3: Fig. S3B). Stimulation of ASH, AVA, AVD, and AVE neurons within the Neural Interactome resulted in oscillatory activity in VA, DA, DD, and AS motor neurons, indicating that oscillations in these neurons were a suitable readout for aversion behaviors (Additional file 3: Fig. S3C). Furthermore, this same circuitry is required for the response to dodecanoic acid (Additional file 3: Fig. S3A). Dodecanoic acid is a toxin produced by the pathogenic bacteria *Streptomyces* [7], thus showing the simulation’s ability to uncover circuits of pathogen avoidance.

**Fig. 2.**
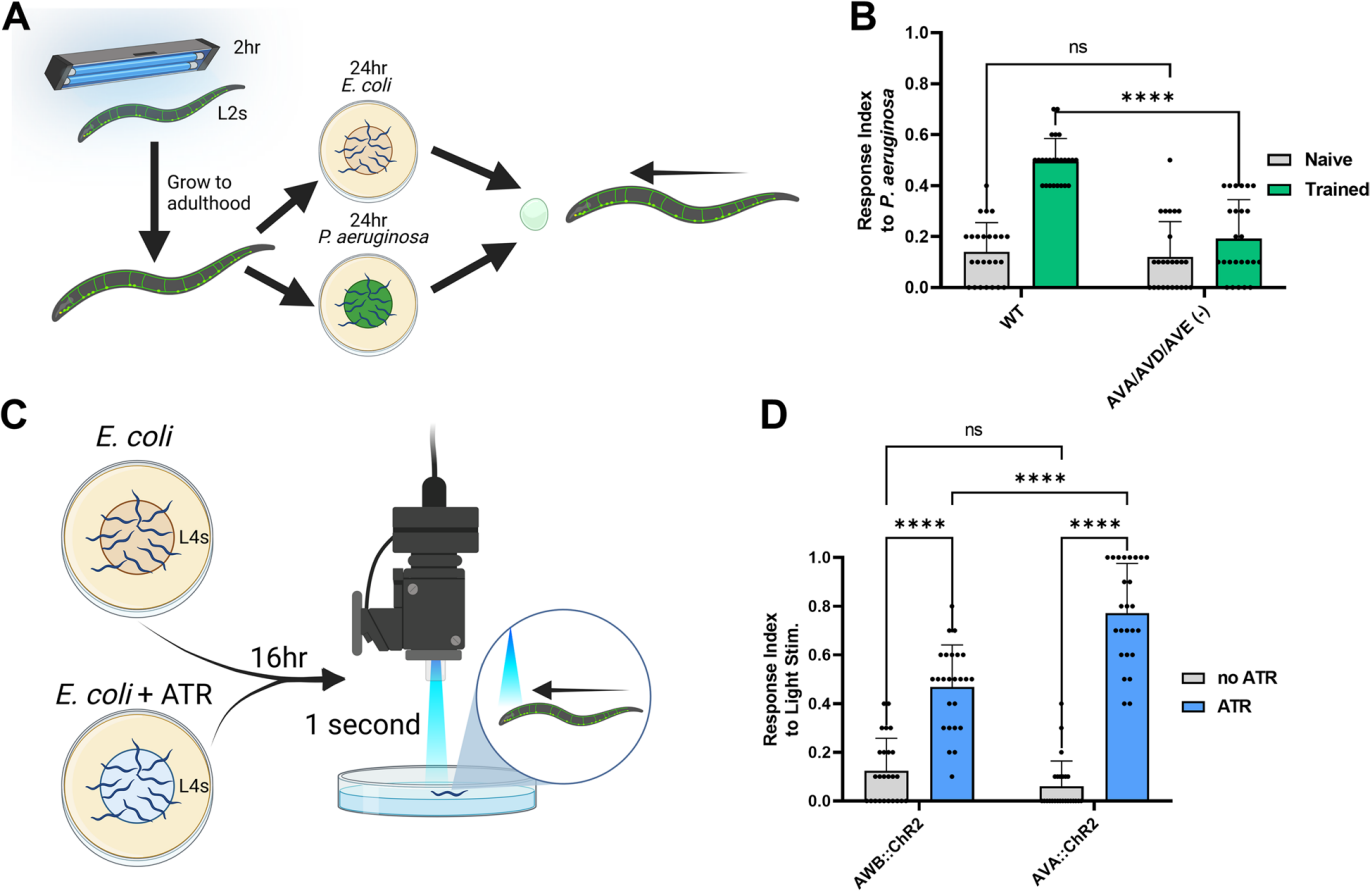
AWB and command interneurons are necessary and sufficient for reflexive aversion. **A** Diagram of light-inducible ablation of neurons without damage to surrounding tissue via a mini singlet oxygen generator (miniSOG), a genetically encoded photosensitizer. L2 wild-type larvae and larvae expressing miniSOG in specific neurons were exposed to continuous blue-light stimulation for 2 h, allowed to reach adulthood, and then tested for reflexive aversion to *P. aeruginosa* both before and after training. **B** Response index to *P. aeruginosa* for both naïve (gray) and trained (green) animals with either no neurons ablated (N2, WT) or AVA/AVD/AVE neurons ablated (ZM7054). **C** Diagram of stimulation of neurons expressing the blue-light-activated cation channel channelrhodopsin-2 (ChR2). Animals were grown to L4 larvae and then transferred to plates with *E. coli* OP50 supplemented with all-trans retinal (ATR). Plates without ATR were used as a control. Animals were allowed to feed overnight for 16 h, and then placed under an epifluorescent microscope fitted with an EGFP filter. Forward crawling animals were stimulated with one second of blue light, and a response recorded if an animal moved backwards during the stimulation. **D** Response index to blue-light stimulation for animals expressing ChR2 in AWB or AVA neurons with no ATR supplementation (gray) and with ATR supplementation (blue). For both **B** and **D**, two-way ANOVA with a subsequent comparison between groups was performed. Error bars depict standard deviation. *N* = 25 (individual dots) for all groups

To experimentally determine whether the CIs AVA, AVD, and AVE are necessary for reflexive aversion to pathogenic bacteria, we utilized a *C. elegans* strain (ZM7054) which allows for blue light-inducible ablation of these neurons without damage to surrounding tissue. Strain ZM7054 carries a mini singlet oxygen generator (miniSOG), a genetically encoded photosensitizer [19]. Wild-type and ZM7054 L2 larvae were exposed to continuous blue-light stimulation for 2 h, allowed to reach adulthood, and then tested for reflexive aversion to *P. aeruginosa* before or after training (Fig. 2A). Using this procedure, we found that animals lacking the CIs failed to display trained reflexive aversion to *P. aeruginosa*, showing a significantly decreased response index compared to wild-type animals (Fig. 2B). We also experimentally tested whether stimulation of either AWB or one of the CIs, AVA, is sufficient to cause reflexive backward locomotion by taking advantage of transgenic lines expressing the blue-light-activated cation channel channelrhodopsin-2 (ChR2) under AWB or AVA specific promoters [19, 28]. Forward crawling animals that had been grown on *E. coli* supplemented with all-trans retinal (ATR, necessary for ChR2 activity and not produced by *C. elegans*) were stimulated with one second of blue light under an epifluorescent microscope, with a response recorded if an animal moved backwards during the stimulation (Fig. 2C). Both AWB::ChR2 and AVA::ChR2 animals displayed significantly increased backward movement responses to blue-light stimulation compared to animals that had not received ATR, with AVA::ChR2 animals showing a stronger response compared to AWB::ChR2 animals (Fig. 2D). Together, these results indicate that AWB and the CIs are necessary for trained reflexive aversion to *P. aeruginosa* and that stimulation of AWB or at least one CI, AVA, is sufficient to cause the backward locomotion that underlies reflexive aversion. This is in line with the interactome simulation (Fig. 1E) and suggests that the interactome is an accurate model of *C. elegans* circuit activity that can be used to predict behavioral outcomes.

### Nervous system simulation predicts that AUA and RMG neurons are part of the AWB neuron-mediated learned reflexive aversion circuit

**Fig. 3.**
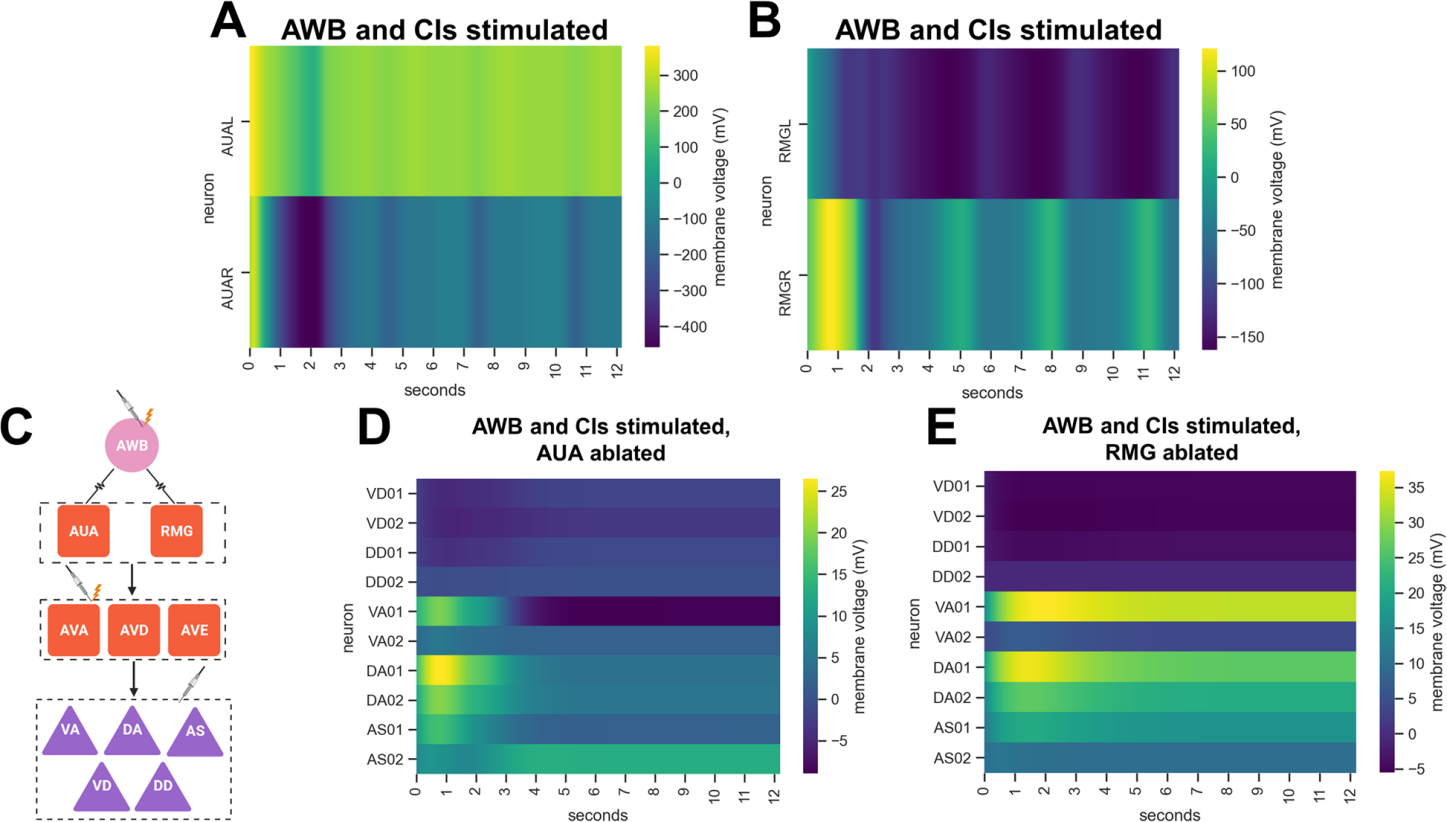
Nervous system simulation predicts that AUA and RMG neurons are in the AWB neuron-mediated learned reflexive aversion circuit. **A** Activity of AUA neurons (rows) upon 5.0 nA stimulation of AWB neurons and 0.9 nA stimulation of the CIs in the Neural Interactome. **B** Activity of RMG neurons (rows) upon 5.0 nA stimulation of AWB neurons and 0.9 nA stimulation of the CIs in the Neural Interactome. **C** Updated circuit diagram and stimulation protocol schematic showing the connections between AWB, AUA, RMG, AVA, AVD, AVE, VA, DA, AS, VD, and DD neurons. The first neuron of each motor neuron class was chosen for ease of visualization. Arrows represent chemical synapses, while jagged lines represent electrical synapses. **D** Activity of motor neurons (rows) upon 5.0 nA stimulation of AWB neurons and 0.9 nA stimulation of the CIs with AUA neurons ablated in the Neural Interactome. **E** Activity of motor neurons (rows) upon 5.0 nA stimulation of AWB neurons and 0.9 nA stimulation of the CIs with RMG neurons ablated in the Neural Interactome

AWB neurons have no direct connection to the motor command AVA, AVD, and AVE interneurons (Fig. 1E). Thus, another layer of interneurons is likely involved in the circuit. Looking at the activity of all the neurons in the interactome model after AWB stimulation, the AUA and RMG neurons stood out as having high activity with oscillations (Fig. 3A, B, and Additional file 4: Fig. S4A). All neurons initially had high peak voltages, with AUAL and RMGR neurons staying highly positive and AUAR and RMGL dropping to low negative voltages. Why there is such a difference in left vs. right neurons is unknown, but the asymmetry could lay the foundations for oscillations in the downstream motor neurons. According to the connectome of *C. elegans*, AUA and RMG form electrical synapses with AWB, and chemically synapse onto the AVA, AVD, and AVE neurons (Fig. 3C [22];). This places them in a prime position to be regulators of the circuit for learned reflexive aversion. Indeed, in silico ablation of either AUA or RMG neurons resulted in the loss of oscillatory activity in VA, DA, VD, DD, and AS motor neurons upon AWB, AVA, AVD, and AVE stimulation (Fig. 3D, E, and Additional file 4: Fig. S4B, and S4C). Other neurons, such as AIB, AVB, and SMB, also bridge the AWB and motor command interneurons, but in silico ablation of these neurons left the motor neuron oscillations intact, while genetic ablation did not affect the trained reflexive aversion to *P. aeruginosa* (Additional file 5: Fig. S5).

### A four-layer circuit underlies pathogen avoidance behaviors and aversion to 2-nonanone

**Fig. 4.**
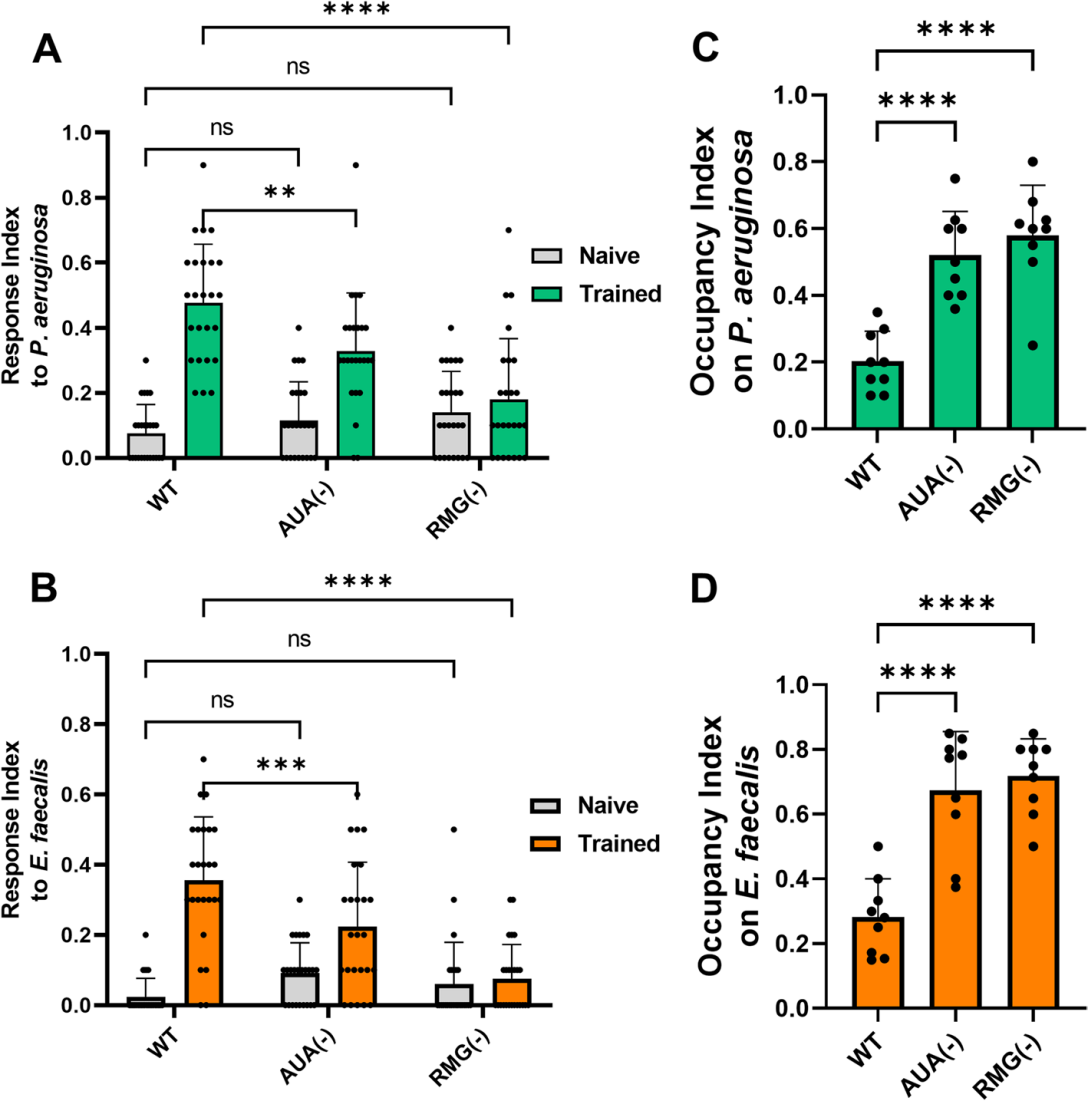
A four-layer circuit underlies pathogen avoidance behaviors. **A** Response index to *P. aeruginosa* for both naïve (gray) and trained (green) animals with either no neurons ablated (WT) or AUA (AY178) or RMG (AY179) neurons ablated. **B** Response index to *E. faecalis* for the same groups as in **A**. For both **A** and **B**, two-way ANOVA with subsequent comparison to naïve or trained WT groups was performed. Error bars depict standard deviation. *N* = 25 (individual dots) for all groups. **C** Occupancy index for *P. aeruginosa* after 24 h for animals with no neurons ablated (WT) or AUA or RMG neurons ablated. (D) Occupancy index for *E. faecalis* after 4 h for the same groups as in **C**. For both **C** and **D**, one-way ANOVA with subsequent comparison to the WT group was performed. Error bars depict standard deviation. *N* = 9 (individual dots) for all groups

To test whether AUA and RMG neurons affected learned reflexive aversion, we genetically ablated AUA and RMG neurons (Additional file 6: Fig. S6) and tested their response to drops of *P. aeruginosa* and *E. faecalis*. Ablation of either AUA or RMG left naïve responses to both bacteria intact, while trained responses showed a significant decrease (Fig. 4A, B). We also measured the occupancy index of animals lacking either AUA or RMG for both *P. aeruginosa* and *E. faecalis* and found that loss of either neuron resulted in increased lawn occupancy (Fig. 4C, D). This indicates that AUA and RMG neurons contribute to both learned reflexive aversion and learned pathogen avoidance of *P. aeruginosa* and *E. faecalis*.

**Fig. 5.**
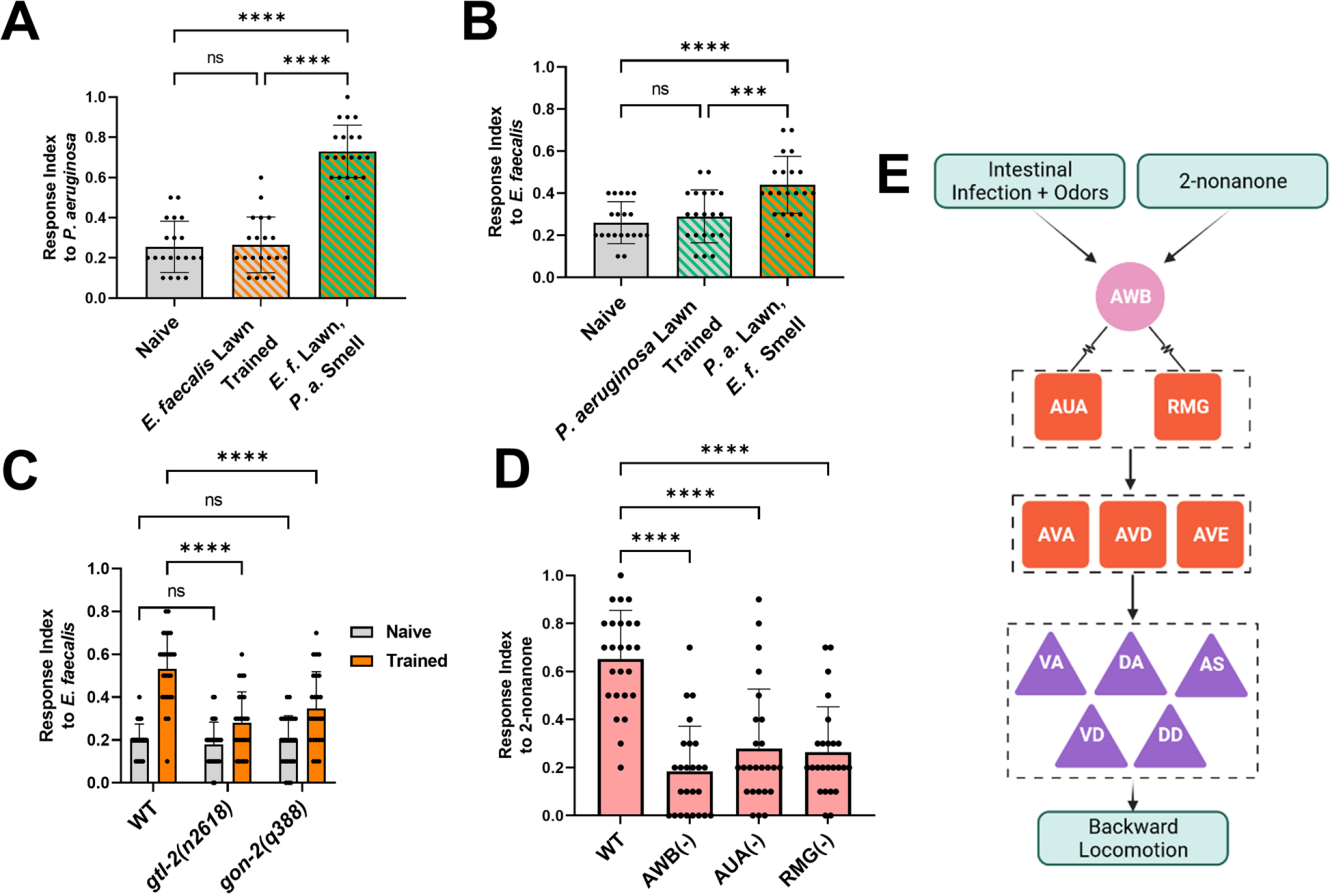
The sensorimotor circuit involves olfaction and regulates aversion to 2-nonanone. **A** Response index to *P. aeruginosa* for wild-type animals either naïve to *P. aeruginosa*, trained on *E. faecalis* lawns, or trained on *E. faecalis* lawns with a lawn of *P. aeruginosa* on the lid, inaccessible to the animals. **B** Response index to *E. faecalis* for wild-type animals either naïve to *E. faecalis*, trained on *P. aeruginosa*, or trained on *P. aeruginosa* lawns with a lawn of *E. faecalis* on the lid. For both **A** and **B**, one-way ANOVA with subsequent comparisons between all groups was performed. Error bars depict standard deviation. *N* = 20 (individual dots) for all groups. **C** Naïve and trained response index to *E. faecalis* for wild-type (WT) and either *gtl-2* or *gon-2* loss-of-function mutants. Two-way ANOVA with subsequent comparison to the WT groups was performed. Error bars depict standard deviation. *N* = 25 (individual dots) for all groups. **D** Response index to 2-nonanone (1:10) for animals with no neuronal ablation (WT) or AWB, AUA, or RMG neurons ablated. One-way ANOVA with subsequent comparison to the WT group was performed. Error bars depict standard deviation. *N* = 25 (individual dots) for all groups. **E** Diagram of the odor-aversion sensorimotor circuit. AWB neurons sense an olfactory cue either from pathogenic bacteria or other repulsive odorants such as 2-nonanone. They pass this signal to AUA and RMG neurons via electrical synapses. AUA and RMG neurons form chemical synapses with AVA, AVD, and AVE command interneurons, which synapse with the motor neurons important for backward locomotion, VA, DA, VD, DD, and AS neurons. These neurons execute the backward locomotion necessary for odor avoidance

We wondered whether the learned reflexive aversion response was specific to the bacteria used to train the animals or if it was a general result of bacterial infection. We cross-tested animals, training them on *E. faecalis* and then testing them with drops of *P. aeruginosa* and vice versa, and found that animals did not show any reflexive aversion (Fig. 5A, B). However, if *E. faecalis* lawns were paired with the odor of *P. aeruginosa* during training, achieved by placing an agar plug of *P. aeruginosa* on the lid of the training plates, animals showed reflexive aversion (Fig. 5A). The same was true for the opposite pairing (Fig. 5B), suggesting that underlying learned reflexive aversion is a process whereby animals associate the odors of the bacteria with infection.

Because we noticed a significant variation in the reflexive aversion of trained animals, we wondered whether varying levels of intestinal distention, which can trigger learned avoidance [10, 25], accounted for the variation in the responses. Intestinal distention on *P. aeruginosa*, measured by either PA14-GFP signal in the intestinal lumen or intestine diameter, correlated with the trained response index to a weak, but significant, amount (Additional file 7: Fig. S7A and B). However, intestinal distention on *E. faecalis* showed no such correlation, perhaps due to the fact that distention was much more prevalent and severe in animals exposed to *E. faecalis* (Additional file 7: Fig. S7C and D). The association of intestinal distention and *E. faecalis* cues requires the TRPM channels GON-2 and GTL-2 [10]. We found that these channels were also required for the learned reflexive aversion to *E. faecalis*, as animals with loss-of-function mutations in the genes encoding for GON-2 and GTL-2 displayed mitigated responses to *E. faecalis* after training (Fig. 5C). Although intestinal distention may not explain the variation in aversion observed, this data suggests that the association of intestinal distention and bacterial cues is required for the trained reflexive aversion to at least *E. faecalis*, indicating that the mechanisms of learned reflexive aversion and learned avoidance share similar connections.

Finally, we wondered whether this circuit was specific to the pathogen response or if it governed a general reflexive aversion to repulsive odors. We found that animals lacking AWB neurons showed reduced reflexive aversion to 2-nonanone, a volatile organic compound that is known to be repulsive to *C. elegans* (Fig. 5D). This confirms previous reports that AWB is the primary senor of 2-nonanone [29, 30]. Importantly, animals lacking either AUA or RMG also showed reduced reflexive aversion to 2-nonanone (Fig. 5D). Altogether, as illustrated in Fig. 5E, these results indicate that learned reflexive aversion involves the formation of an association between intestinal infection and bacterial odors, with the behavior executed by a four-layer neural circuit composed of AWB olfactory neurons electrically synapsed to AUA and RMG interneurons, which themselves are connected chemically to the motor-command interneurons AVA, AVD, and AVE. These interneurons control the motor neurons, VA, DA, VD, DD, and AS, which execute backward locomotion. Backward locomotion and the underlying circuit seem to be crucial for learned reflexive aversion to *P. aeruginosa* and *E. faecalis*, learned avoidance of lawns of these pathogens, and aversion to a repulsive volatile organic compound, 2-nonanone, in *C. elegans*. Taken together, the results implicate the sensorimotor circuit in the control of an innate reflexive aversion to a repulsive odor and a learned aversion to pathogenic bacteria.

## Discussion

The discovery of a sensorimotor circuit involved in learned reflexive aversion to the pathogenic bacteria *P. aeruginosa* and *E. faecalis* and naïve aversion to 2-nonanone demonstrates the utility of the *C. elegans* Neural Interactome as a tool for hypothesis generation. AUA and RMG neurons had not previously been implicated in either pathogen avoidance or aversion to 2-nonanone. Interestingly, both had been implicated in the regulation of social feeding behavior [31, 32]. AUA is a synaptic target of URX, and expression of *npr-1* in *npr-1(ad609)* mutants in AQR, PQR, URX, and AUA neurons results in suppression of aggregation and bordering behaviors [31]. RMG is at the center of a gap junction hub-and-spoke circuit, connected to many sensory neurons, including ASK, URX, ASH, ADL, and AWB [32]. High activity in RMG is essential for all aspects of social behavior, including aggregation, bordering, and, with input from ASK neurons, attraction to hermaphrodite pheromones. The innexin gene *unc-9* was shown to be required in RMG neurons to drive social behavior [33]. As AWB and AUA neurons also express *unc-9* [34], it is possible that *unc-9*-based gap junctions are also required for AWB-mediated pathogen and odor avoidance behaviors.

A crucial step to study sensorimotor circuits necessary for learned pathogen avoidance was to find a behavior that matched the seconds-long timescale necessary to perform simulations. Learned pathogen avoidance takes hours and was therefore not a suitable behavior. The discovery that *C. elegans* avoids drops of *P. aeruginosa* and *E. faecalis* within seconds, following hour-long pre-exposure on bacterial lawns, allowed us to match the behavior and the simulated data from the interactome on similar timescales. Ultimately, the neurons involved in learned reflexive aversion and learned pathogen avoidance were the same for both *P. aeruginosa* and *E. faecalis*, with AUA and RMG neurons required for both (Fig. 4). Thus, we speculate that learned reflexive aversion to pathogenic bacteria is a crucial component of the general learned pathogen avoidance behavior. In general avoidance, the reflex to avoid pathogenic bacteria is most likely countered by attraction to the bacteria as a food source. This attraction is likely driven by AWC neurons, which are known to shape the olfactory response to pathogenic bacteria and food odors [9, 35]. We found that AWC neurons were required for the learned reflexive aversion response, further implicating them in the associative learning process required for avoidance of *P. aeruginosa* and *E. faecalis* (Fig. 1B, C). The cross-training experiments performed with the two pathogens make clear that this process is driven by olfaction (Fig. 5A, B). Exactly how this learning takes place and what the changes in the neural dynamics are that allow for a shift from attraction to aversion over time remains unclear, though modulation via serotonin likely plays a role [9, 13]. ASI and ASE neurons also seem to play a role in the learned aversion to *P. aeruginosa* and *E. faecalis*, respectively (Fig. 1B, C). The simulated data suggest that such a role is not through the engagement of the backward-movement motor neuron circuitry, as stimulation of AWC, ASE, or ASI neurons does not lead to strong oscillations in the motor neurons (Additional file 2: Fig. S2). This is in contrast to the stimulation of AWB neurons, which induces motor neuron oscillations (Fig. 1E and Additional file 1: Fig. S1). What bacterial cue actually stimulates the AWB neurons remains unknown, though it is likely a bacterial odor (or blend of odors). This could be an initially attractive odor that becomes aversive with bacterial infection, or an innately aversive odor such as 1-undecene [6].

As genetic ablation of specific neurons may have potential off-target effects, it was important to confirm the involvement of neurons revealed by ablation experiments through additional methods. To this end, we utilized an optogenetic approach and showed that activating AWB or AVA neurons was sufficient to induce backward movement indicative of an aversive response (Fig. 2C, D). Additional approaches, such as expressing histamine-gated chloride channels in individual neurons, would further confirm the necessity of these neurons in the aversive circuit. Related to this, while the simplest explanation of the data presented here is a linear circuit going from AWB to AUA/RMG to AVA/AVD/AVE to the motor neurons, we cannot completely rule out a situation where AWB and AUA/RMG or AVA/AVD/AVE act in parallel and lead to the same behavioral outcome. However, as AUA, RMG, AVA, AVD, and AVE are not sensory neurons, they are unlikely to be directly stimulated by bacterial cues. Instead, there would have to be a sensory neuron upstream of them that acts as the bacterial cue sensor. The experiments presented here suggest that AWB neurons fulfill this role in the context of aversive bacterial odors, while in situations such as the response to touch or dodecanoic acid, ASH neurons fulfill this role (Fig. S3). It is also possible that AWB neurons form a circuit with neurons other than AUA/RMG-AVA/AVD/AVE that are necessary for the aversion behavior. However, the optogenetic experiments (Fig. 2C, D), ablation of AVA, AVD, and AVE leading to decreased aversion (Fig. 2A, B), and ablation of AIB or AVB not affecting aversion (Additional file 5: Fig. S5), suggest otherwise. At this stage, we cannot completely rule out other circuit possibilities.

While the interactome allowed us to accurately predict which neurons would be necessary for pathogen avoidance and 2-nonanone aversion, the actual neural dynamics at play in these behaviors may be quite different. An important next step would be to experimentally uncover the dynamics at play and compare them to the interactome model. This would allow for further refinement of the model and would be possible using tools such as NeuroPAL, which allows for neuronal identification of all *C. elegans* neurons via a multicolor fluorescence map and genetically encoded calcium indicators [36]. These tools will allow us to distinguish whether the subtleties in neuronal activity predicted by the simulation (for example, differences in peak membrane voltages, phases of motor neuron oscillations, and AUA/RMG left vs. right neuron activity) might be artifacts of the simulation or important components of the circuit that shapes the aversion response. Further combinations of experimental manipulation and computational modeling will be crucial in deepening our understanding of behaviorally relevant neural circuits.

## Conclusions

We have studied the neural circuitry underlying a trained aversive backward locomotion response to pathogenic bacteria following infection. Using whole-brain simulation and functional assays, we uncovered a four-layer circuit composed of AWB olfactory neurons, AUA and RMG interneurons, AVA, AVE, and AVD command interneurons, and a motor neuron layer that controls pathogen aversion. This response involves intestinal distention and, for *E. faecalis*, requires expression of TRPM channels in the intestine and excretory system. This identification of a complete sensorimotor circuit for pathogen avoidance demonstrates the utility of combining computational connectomics and functional assays to uncover new neuronal regulators of behavior.

## Methods

### Bacterial strains

The following bacterial strains were used: *Enterococcus faecalis* OG1RF, *E. faecalis* OG1RF-GFP, *Escherichia coli* OP50, *Pseudomonas aeruginosa* PA14, and *P. aeruginosa* PA14-GFP. *E. coli* and *P. aeruginosa* bacterial strains were grown in Luria-Bertani (LB) broth at 37 °C, while the *E. faecalis* was grown in brain-heart infusion (BHI) broth at 37 °C.

### *C. elegans* strains and maintenance

The following *C. elegans* strains used in this study were obtained from the CGC: N2 (WT strain), CZ9957 *gtl-2(n2618)*, EJ1158 *gon-2(q388)*, JN1713 peIs1713 [*sra-6p::mCasp-1 + unc-122p::mCherry*], JN1715 peIs1715 [*str-1p::mCasp-1 + unc-122p::GFP*], JN578 peIs578 [*npr-9p::casp1* + *npr-9p::Venus* + *unc-122p::*mCherry], NY2078 ynIs78 [*flp-8p::GFP*], NY2087 ynIs87 [*flp-21p::GFP*], PR680 *che-1(p680)*, PY7502 oyIs85 [*ceh-36p::TU#813 + ceh-36p::TU#814 + srtx-1p::GFP + unc-122p:DsRed*], PY7505 oyIs84 [*gpa-4p::TU#813 + gcy-27p::TU#814 + gcy-27p::GFP + unc-122p::DsRed*], and ZM6804 hpIs270 [*rig-3p::FRT::stop::FRT::ChR2(H134R)::wCherry* + *nmr-1p::FLP* + *lin-15(+)*], ZM7297 hpIs331 [*lgc55Bp::tomm20::miniSOG::SL2::RFP* + *lin-15(+)*]. The following transgenic strains were generated for this study using standard microinjection protocols [10]: AY178 ynIs78 [*flp-8p::GFP*]; *flp-8p::ced-3 (p15)::nz + flp-32::cz::ced-3 (p17) + unc-122p::rfp* and AY179 ynIs87 [*flp-21p::GFP*]; *flp-21p::ced-3 (p15)::nz + ncs-1p::cz::ced-3 (p17) + unc-122p::rfp*. AWB::ChR2 (raxIs15 [*str-1p::ChR2::sl2gfp*] was a gift from Meng C. Wang’s laboratory. *C. elegans* hermaphrodites were maintained on *E. coli* OP50 at 20 °C.

### Construction of neuronal ablation strains

The transgenic strains AY178 (AUA ablation) and AY179 (RMG ablation) were generated by first constructing pJL12 (pPD95.75 *flp-8p::ced-3 (p15)::nz*), pJL13 (pPD95.75 *flp-32p::cz::ced-3 (p17)*), pJL14 (pPD95.75 *flp-21p::ced-3 (p15)::nz*), and pJL15 (pPD95.75 *ncs-1p:: cz::ced-3 (p17)*) plasmids. The pJL12 plasmid was constructed by cloning a 2019 bp upstream-promoter region of the *flp-8* gene into a *ced-3 (p15)::nz* backbone vector, via SphI-BamHI restriction sites. The pJL13 plasmid was constructed by cloning a 2085 bp upstream-promoter region of the *flp-32* gene into a cz::ced-3 (p17) backbone vector, via SphI-BamHI restriction sites. The pJL14 plasmid was constructed by cloning a 4109 bp upstream-promoter region of the *flp-21* gene into a *ced-3 (p15)::nz* backbone vector, via SphI-BamHI restriction sites. The pJL15 plasmid was constructed by cloning a 3116 bp upstream-promoter region of the *ncs-1* gene into a *cz::ced-3 (p17)* backbone vector, via SphI-BamHI restriction sites. recCaspase plasmids were a gift from Martin Chalfie, Addgene plasmids # 16080 and # 16081, Addgene, MA [37]. A cocktail of pJL12 (10ng/μl), pJL13 (10ng/μl), co-injection marker *unc-122p::RFP* (50ng/μl), and empty vector PUC18 (50ng/μl) plasmids was co-injected into NY2078 animals to generate AY178 animals. A cocktail of pJL14 (10ng/μl), pJL15 (10ng/μl), co-injection marker *unc-122p::RFP* (50ng/μl), and empty vector PUC18 (50ng/μl) plasmids was co-injected into NY2087 animals to generate AY179 animals. Transgenic animals showing successful ablation of AUA or RMG neurons were selected and used for further assays. Plasmids were maintained as extrachromosomal arrays.

### Dry-drop assay

The dry-drop assay was carried out as previously described [7]. Using a capillary, a drop of the appropriate stimulus was placed on a dry SK plate (for SDS, dodecanoic acid, 2-nonanone, and *P. aeruginosa*) or BHI plate (for *E. faecalis*) in front of a forward-moving animal. A response was counted if an animal initiated backward movement upon encountering the dried drop. Response Index = N_responses_/N_drops_. Ten drops were used per animal, and a total of 25 animals of each strain were used for each experiment. The stimuli were prepared as follows: 0.6mM SDS, 1mM dodecanoic acid, 1:10 2-nonanone, and a 5–6 h liquid culture of either *P. aeruginosa* or *E. faecalis.*

### Lawn avoidance assays

Lawn avoidance assays were carried out as previously described [10]. Briefly, bacterial cultures were grown by inoculating *P. aeruginosa* or *E. faecalis* colonies into 2 mL of either LB or BHI broth, respectively, and growing them for 5–6 h on a shaker at 37 °C. Then, 20 μL of the culture was plated onto the center of 3.5-cm-diameter BHI or standard slow-killing (SK) plates (modified NGM agar plates [0.35% instead of 0.25% peptone]). The plates were then incubated overnight at 37 °C. The plates were cooled to room temperature for at least 30 min before seeding with animals. Synchronized young gravid adult hermaphroditic animals grown on *E. coli* OP50 were washed with M9 buffer and transferred outside the bacterial lawns, and the number of animals on and off the lawns were counted at 24 h for *P. aeruginosa* and 4 h for *E. faecalis*. Three 3.5-cm-diameter plates were used per trial in every experiment. Occupancy index was calculated as *N*_on lawn_/*N*_total_.

### Aversive training

Training plates of 3.5-cm-diameter containing either *P. aeruginosa* on SK agar or *E. faecalis* on BHI agar were made as described above for avoidance assays. Young gravid adult hermaphroditic animals grown on *E. coli* OP50 were washed with M9 and transferred to the training plates and allowed to roam for 24 h on *P. aeruginosa* and 4 h on *E. faecalis.* They were then transferred to the appropriate assay plates. For cross-training with odors (Fig. 2D, E), agar plugs with bacterial lawns were cut from growth plates and transferred to the lids of the training plates with the appropriate training bacteria, as previously described [10].

### Neuronal ablation by miniSOG

L2 wild-type larvae and larvae expressing miniSOG in specific neurons were exposed to continuous blue-light stimulation in a custom lightbox fitted with blue LED strips at 25 °C for 2 h. Animals were then removed from the lightbox and allowed to reach adulthood at 20 °C. Animals were then tested for reflexive aversion to *P. aeruginosa* both before and after training using the methods described above.

### Optogenetic stimulation of neurons

Animals expressing the blue-light-activated cation channel channelrhodopsin-2 (ChR2) in specific neurons were grown to L4 larvae and then transferred to plates with *E. coli* OP50 supplemented with all-trans retinal (ATR; 100μM working solution). Plates without ATR were used as a control. Animals were allowed to feed overnight for 16 h, and then placed under an epifluorescent microscope equipped with an EGFP filter. Forward crawling animals were stimulated with one second of blue light, and a positive response was recorded if an animal moved backward during the stimulation.

### Imaging and quantification

Fluorescence imaging was carried out as described previously [10]. Briefly, the animals were anesthetized using an M9 salt solution containing 50 mM sodium azide and mounted onto 2% agar pads. The animals were then visualized using a Leica M165 FC fluorescence stereomicroscope. For quantification of intestinal lumen distention, brightfield images were acquired at 24 h for *P. aeruginosa* and 4 h for *E. faecalis* using the Leica LAS v4.6 software, and the diameter of the intestinal lumen was measured using ImageJ software. For quantification of fluorescent bacteria, fluorescent images were acquired using the Leica LAS v4.6 software, and ImageJ software was used to first draw a region of interest around the bacteria in the intestines of animals and then to measure fluorescence intensity in the region.

### Neural Interactome simulations and visualization

Simulations of the *C. elegans* nervous system were carried out using a local copy of the Neural Interactome, pulled from https://github.com/shlizee/C-elegans-Neural-Interactome. Simulations were run for at least 12 s and neuronal activity for each simulation was saved into a numpy file. Simulated data was loaded directly with the Python Numpy package into a Jupyter Notebook and converted to a Pandas DataFrame. Python scripts were written to visualize individual neuronal activity over time as rows on a heatmap and graphs were generated using the Matplotlib and Seaborn libraries. The first two neurons of the VD, DD, VA, DA, and AS motor neuron classes were chosen as representatives of each class to aid visualization.

### Statistical analysis

The statistical analysis was performed with Prism 9 (GraphPad). All bar graphs depict the mean of the population, with individual dots representing individual trials. Error bars represent the standard deviation of the population. For *x*-*y* correlation plots, individual dots represent measurements for individual animals, and the red line depicts a simple linear regression. All experiments were performed in triplicate on at least three separate days, resulting in the sample sizes listed in the figure legends. One- or two-way ANOVA with subsequent group comparisons were performed as indicated in the figure legends. In the figures, ns denotes not significant, and asterisks (*) denote statistical significance as follows: *≤0.05; **p ≤ 0.01; ***p ≤ 0.001; ****p ≤ 0.0001, as compared with the appropriate controls.

## Supplementary Information

**Additional file 1: Fig. S1.** Motor neurons show oscillations upon AWB and command interneuron simulated stimulation. (A) The same data as in Fig. 1D presented as waveforms. (B) The same data as in Fig. 1E presented as waveforms. For each scenario, the first neuron from each motor neuron subclass is shown for ease of visualization.

**Additional file 2: Fig. S2.** AWC, ASI, and ASE neurons do not lead to oscillations in motor neurons important for backward locomotion. Heatmaps (above) and waveforms (below) of VD, DD, VA, DA, and AS motor neuron activity in the Neural Interactome upon 0.9 nA stimulation of the CIs and 5.0 nA stimulation of AWC (A), ASI (B), and ASE (C).

**Additional file 3: Fig. S3.** The response to SDS and dodecanoic acid are ASH mediated and can be modeled in the Neural Interactome. (A) Response index to buffer, 0.6 mM SDS, or 1mM dodecanoic acid for animals with no neurons ablated (WT, red) or ASH neurons ablated (ASH(-), purple). Two-way ANOVA with subsequent comparison to the WT groups was performed. Error bars depict standard deviation. *N* = 15 for buffer, 10 for SDS, and 25 for dodecanoic acid (individual dots). (B) Diagram of the circuit for reflexive aversion to SDS and dodecanoic acid. Arrows represent chemical synapses, while jagged lines represent electrical synapses. (C) Heatmap (above) and waveforms (below) of activity for the motor neurons VD, DD, VA, DA, and AS (rows) upon 5.0 nA stimulation of ASH neurons and 0.9 nA stimulation of the CIs in the Neural Interactome.

**Additional file 4: Fig. S4.** AUA and RMG neurons are required for motor neuron oscillations upon AWB and command interneuron stimulation in simulations of the *C. elegans* nervous system. (A) The same data as in Fig. 3A and B presented as waveforms. (B) The same data as in Fig. 3D presented as waveforms. (C) The same data as in Fig. 3E presented as waveforms.

**Additional file 5: Fig. S5.** AIB, AVB, or SMB neuron ablation does not diminish oscillations in backward locomotion-associated motor neurons. Heatmaps (left) and waveforms (right) of activity of motor neurons (rows) upon 5.0 nA stimulation of AWB neurons and 0.9 nA stimulation of the CIs with AIB (A), AVB (B), or SMB (C) neurons ablated in the Neural Interactome. (D) Response index to *P. aeruginosa* for both naïve (gray) and trained (green) animals with either no neurons ablated (N2, WT) or AIB (JN578) or AVB (ZM7297) neurons ablated. For ZM7297 animals, the miniSOG ablation protocol was followed as in Fig. 2A. Two-way ANOVA with subsequent comparison to naïve or trained WT groups was performed. Error bars depict standard deviation. *N* = 25 (individual dots) for all groups.

**Additional file 6: Fig. S6.** Genetic ablation of AUA and RMG neurons. (A) Representative fluorescent micrographs of NY2078 ynIs78 [*flp-8p::GFP*] (left) and AY178 ynIs78 [*flp-8p::GFP*]; *flp-8p::ced-3 (p15)::nz + flp-32::cz::ced-3 (p17) + unc-122p::rfp* (right) animals. (B) Representative fluorescent micrographs of NY2087 ynIs87 [*flp-21p::GFP* (left) and AY179 ynIs87 [*flp-21p::GFP*]; *flp-21p::ced-3 (p15)::nz + ncs-1p::cz::ced-3 (p17) + unc-122p::rfp* (right) animals. White, filled arrows point to intact AUA or RMG neurons, while white, unfilled arrows point to the lack of AUA or RMG neurons. GFP-positive neurons were counted for both intact and ablated animals (pink circles), showing that only the targeted neurons were ablated, with other neurons left intact. AUA intact: 4 GFP neurons; AUA ablated: 2 GFP neurons; RMG intact: 15 GFP neurons; RMG ablated: 13 neurons. Scale bars are 50 μm.

**Additional file 7: Fig. S7.** Correlation of intestinal distention and learned reflexive aversion for *P. aeruginosa* but not *E. faecalis* exposure. (A) PA14::GFP relative fluorescence in the intestine (x-axis) and the trained response index to *P. aeruginosa* (y-axis) were measured in individual animals (dots), and linear regression was performed (red line). (B) Same as A but with intestinal diameter on *P. aeruginosa* (x-axis). (C) Same as A but with *E. faecalis.* (D) Same as B but with *E. faecalis*. R^2^ and P-values are shown next to linear regression lines in red.

**Additional file 8: Data.** Combined raw data, with each figure on a separate spreadsheet.

## Data Availability

All data generated or analyzed during this study are included in this published article and its supplementary information files.

## References

[1] Schaller M, Park JH (2011). The behavioral immune system (and why it matters). Curr Dir Psychol Sci.

[2] Regenbogen C, Axelsson J, Lasselin J, Porada DK, Sundelin T, Peter MG (2017). Behavioral and neural correlates to multisensory detection of sick humans. Proc Natl Acad Sci.

[3] Stensmyr MC, Dweck HKM, Farhan A, Ibba I, Strutz A, Mukunda L (2012). A conserved dedicated olfactory circuit for detecting harmful microbes in *drosophila*. Cell..

[4] Meisel JD, Kim DH (2014). Behavioral avoidance of pathogenic bacteria by *Caenorhabditis elegans*. Trends Immunol.

[5] Pradel E, Zhang Y, Pujol N, Matsuyama T, Bargmann CI, Ewbank JJ (2007). Detection and avoidance of a natural product from the pathogenic bacterium *Serratia marcescens* by *Caenorhabditis elegans*. Proc Natl Acad Sci U S A.

[6] Prakash D, Ms A, Radhika B, Venkatesan R, Chalasani SH, Singh V (2021). 1-Undecene from *Pseudomonas aeruginosa* is an olfactory signal for flight-or-fight response in *Caenorhabditis elegans*. EMBO J.

[7] Tran A, Tang A, O’Loughlin CT, Balistreri A, Chang E, Coto Villa D (2017). *C. elegans* avoids toxin-producing *Streptomyces* using a seven transmembrane domain chemosensory receptor. Hobert O, editor. eLife.

[8] Styer KL, Singh V, Macosko E, Steele SE, Bargmann CI, Aballay A (2008). Innate immunity in *Caenorhabditis elegans* is regulated by neurons expressing NPR-1/GPCR. Science..

[9] Zhang Y, Lu H, Bargmann CI (2005). Pathogenic bacteria induce aversive olfactory learning in *Caenorhabditis elegans*. Nature..

[10] Filipowicz A, Lalsiamthara J, Aballay A (2021). TRPM channels mediate learned pathogen avoidance following intestinal distention. Sengupta P, Portman D, Portman D, O’donnell M, editors. eLife.

[11] Meisel JD, Panda O, Mahanti P, Schroeder FC, Kim DH (2014). Chemosensation of bacterial secondary metabolites modulates neuroendocrine signaling and behavior of *C. elegans*. Cell..

[12] Kaletsky R, Moore RS, Vrla GD, Parsons LR, Gitai Z, Murphy CT. *C. elegans* interprets bacterial non-coding RNAs to learn pathogenic avoidance. Nature. 2020;586:445–51. 10.1038/s41586-020-2699-5 PMC8547118 32908307

[13] Ha H, Hendricks M, Shen Y, Gabel CV, Fang-Yen C, Qin Y (2010). Functional organization of a neural network for aversive olfactory learning in *Caenorhabditis elegans*. Neuron..

[14] Reddy KC, Hunter RC, Bhatla N, Newman DK, Kim DH (2011). *Caenorhabditis elegans* NPR-1–mediated behaviors are suppressed in the presence of mucoid bacteria. Proc Natl Acad Sci U S A.

[15] Reddy KC, Andersen EC, Kruglyak L, Kim DH (2009). A polymorphism in *npr-1* is a behavioral determinant of pathogen susceptibility in *C. elegans*. Science..

[16] Hao Y, Yang W, Ren J, Hall Q, Zhang Y, Kaplan JM (2018). Thioredoxin shapes the *C. elegans* sensory response to *pseudomonas* produced nitric oxide. eLife..

[17] Brandt JP, Ringstad N (2015). Toll-like receptor signaling promotes development and function of sensory neurons required for a *C. elegans* pathogen-avoidance behavior. Curr Biol.

[18] Chen Z, Hendricks M, Cornils A, Maier W, Alcedo J, Zhang Y (2013). Two insulin-like peptides antagonistically regulate aversive olfactory learning in *C. elegans*. Neuron..

[19] Gao S, Guan SA, Fouad AD, Meng J, Kawano T, Huang YC (2018). Excitatory motor neurons are local oscillators for backward locomotion. eLife..

[20] Haspel G, O’Donovan MJ, Hart AC (2010). Motoneurons dedicated to either forward or backward locomotion in the nematode *Caenorhabditis elegans*. J Neurosci.

[21] Chalfie M, Sulston JE, White JG, Southgate E, Thomson JN, Brenner S (1985). The neural circuit for touch sensitivity in *Caenorhabditis elegans*. J Neurosci.

[22] Cook SJ, Jarrell TA, Brittin CA, Wang Y, Bloniarz AE, Yakovlev MA (2019). Whole-animal connectomes of both *Caenorhabditis elegans* sexes. Nature..

[23] Sarma GP, Lee CW, Portegys T, Ghayoomie V, Jacobs T, Alicea B (2018). OpenWorm: overview and recent advances in integrative biological simulation of *Caenorhabditis elegans*. Philos Transact Royal Soc B: Biol Sci.

[24] Kim J, Leahy W, Shlizerman E. Neural interactome: interactive simulation of a neuronal system. Front Comput Neurosci. 2019;13:8. 10.3389/fncom.2019.00008 PMC6425397 30930759

[25] Singh J, Aballay A (2019). Intestinal infection regulates behavior and learning via neuroendocrine signaling. Iino Y, VijayRaghavan K, Irazoqui J, editors. eLife.

[26] Wen Q, Gao S, Zhen M (2018). *Caenorhabditis elegans* excitatory ventral cord motor neurons derive rhythm for body undulation. Philos Transact Royal Soc B: Biol Sci.

[27] Hilliard MA, Bargmann CI, Bazzicalupo P (2002). *C. elegans* responds to chemical repellents by integrating sensory inputs from the head and the tail. Curr Biol.

[28] Sowa JN, Mutlu AS, Xia F, Wang MC (2015). Olfaction modulates reproductive plasticity through neuroendocrine signaling in *Caenorhabditis elegans*. Curr Biol.

[29] Troemel ER, Kimmel BE, Bargmann CI (1997). Reprogramming chemotaxis responses: sensory neurons define olfactory preferences in *C. elegans*. Cell..

[30] Bargmann CI, Hartwieg E, Horvitz HR (1993). Odorant-selective genes and neurons mediate olfaction in *C. elegans*. Cell..

[31] Coates JC, de Bono M (2002). Antagonistic pathways in neurons exposed to body fluid regulate social feeding in *Caenorhabditis elegans*. Nature..

[32] Macosko EZ, Pokala N, Feinberg EH, Chalasani SH, Butcher RA, Clardy J (2009). A hub-and-spoke circuit drives pheromone attraction and social behavior in *C. elegans*. Nature..

[33] Jang H, Levy S, Flavell SW, Mende F, Latham R, Zimmer M (2017). Dissection of neuronal gap junction circuits that regulate social behavior in *Caenorhabditis elegans*. Proc Natl Acad Sci U S A.

[34] Altun ZF, Chen B, Wang ZW, Hall DH (2009). High resolution map of *Caenorhabditis elegans* gap junction proteins. Dev Dyn.

[35] Harris G, Shen Y, Ha H, Donato A, Wallis S, Zhang X (2014). Dissecting the signaling mechanisms underlying recognition and preference of food odors. J Neurosci.

[36] Yemini E, Lin A, Nejatbakhsh A, Varol E, Sun R, Mena GE (2021). NeuroPAL: a multicolor atlas for whole-brain neuronal identification in *C. elegans*. Cell..

[37] Chelur DS, Chalfie M (2007). Targeted cell killing by reconstituted caspases. Proc Natl Acad Sci.

