# Supplementary figures and images for: Dissection of a sensorimotor circuit underlying pathogen aversion in C. elegans
Source: BMC Biol. 2022 Oct 8;20:229. doi: 10.1186/s12915-022-01424-x (PMC9548130; doi:10.1186/s12915-022-01424-x)

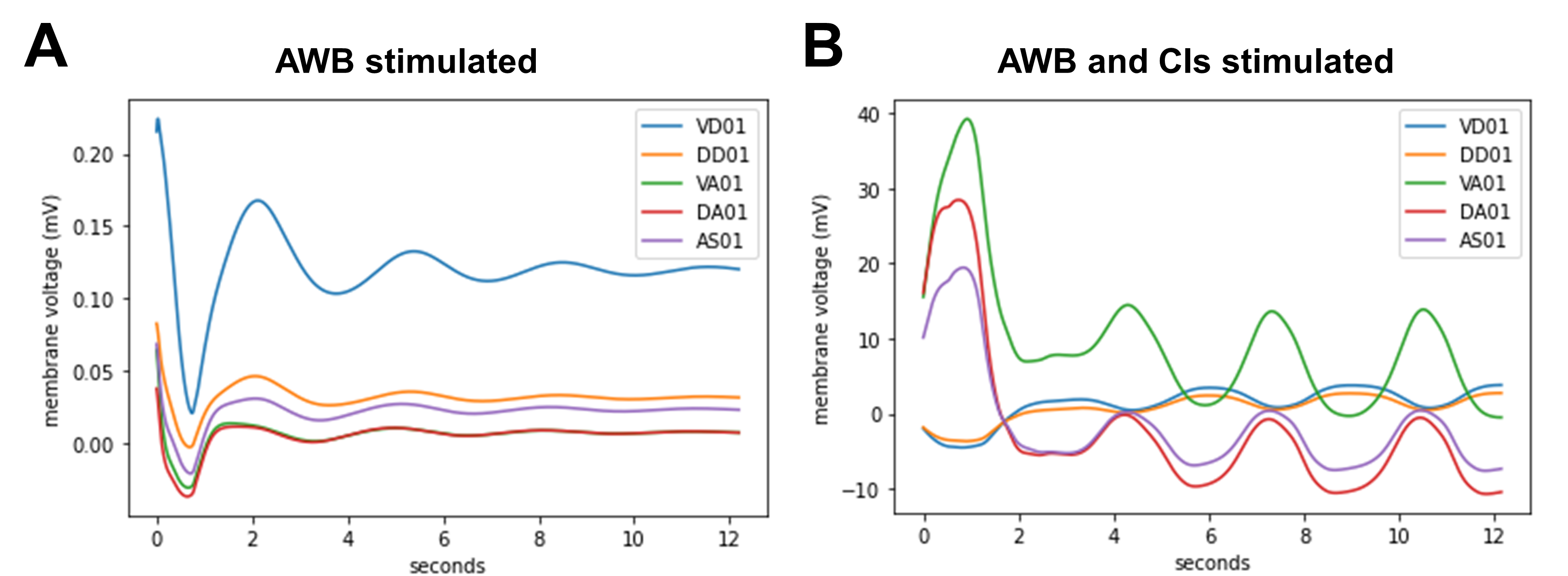

Supplement: Supplementary file 1 — Additional file 1: Fig. S1. Motor neurons show oscillations upon AWB and command interneuron simulated stimulation. (A) The same data as in Fig. 1D presented as waveforms. (B) The same data as in Fig. 1E presented as waveforms. For each scenario, the first neuron from each motor neuron subclass is shown for ease of visualization. [file 12915_2022_1424_MOESM1_ESM.tif]

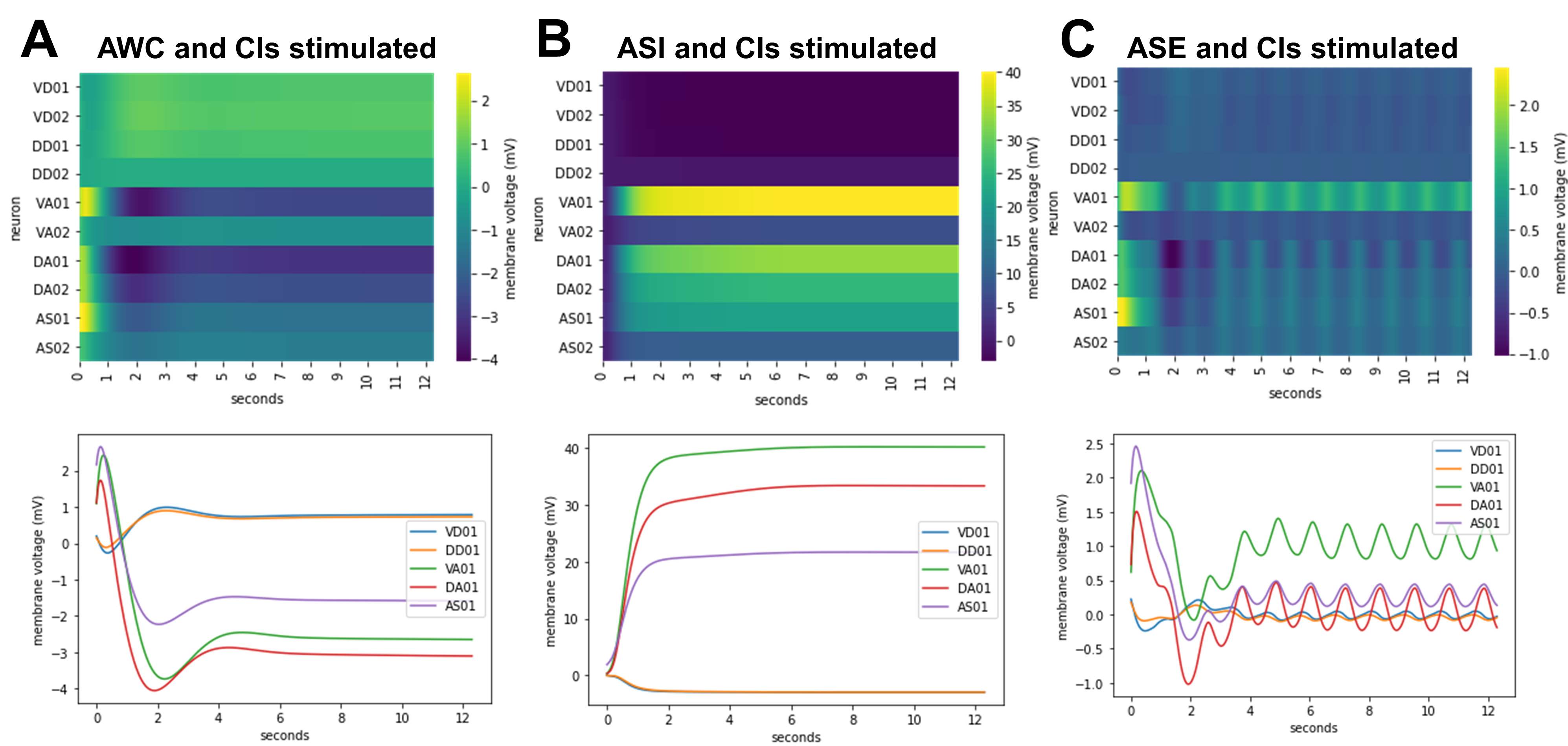

Supplement: Supplementary file 2 — Additional file 2: Fig. S2. AWC, ASI, and ASE neurons do not lead to oscillations in motor neurons important for backward locomotion. Heatmaps (above) and waveforms (below) of VD, DD, VA, DA, and AS motor neuron activity in the Neural Interactome upon 0.9 nA stimulation of the CIs and 5.0 nA stimulation of AWC (A), ASI (B), and ASE (C). [file 12915_2022_1424_MOESM2_ESM.tif]

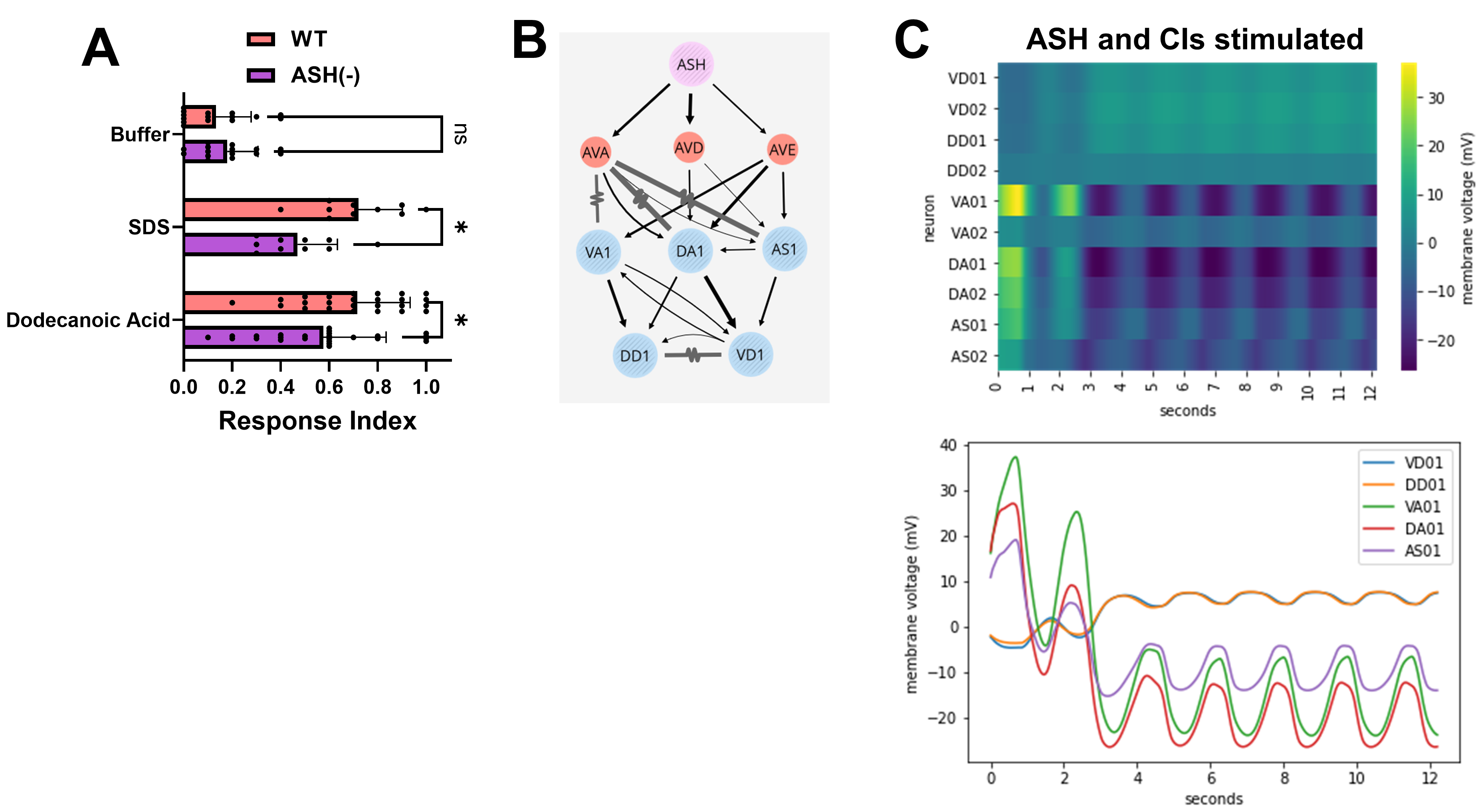

Supplement: Supplementary file 3 — Additional file 3: Fig. S3. The response to SDS and dodecanoic acid are ASH mediated and can be modeled in the Neural Interactome. (A) Response index to buffer, 0.6 mM SDS, or 1mM dodecanoic acid for animals with no neurons ablated (WT, red) or ASH neurons ablated (ASH(-), purple). Two-way ANOVA with subsequent comparison to the WT groups was performed. Error bars depict standard deviation. N = 15 for buffer, 10 for SDS, and 25 for dodecanoic acid (individual dots). (B) Diagram of the circuit for reflexive aversion to SDS and dodecanoic acid. Arrows represent chemical synapses, while jagged lines represent electrical synapses. (C) Heatmap (above) and waveforms (below) of activity for the motor neurons VD, DD, VA, DA, and AS (rows) upon 5.0 nA stimulation of ASH neurons and 0.9 nA stimulation of the CIs in the Neural Interactome. [file 12915_2022_1424_MOESM3_ESM.tif]

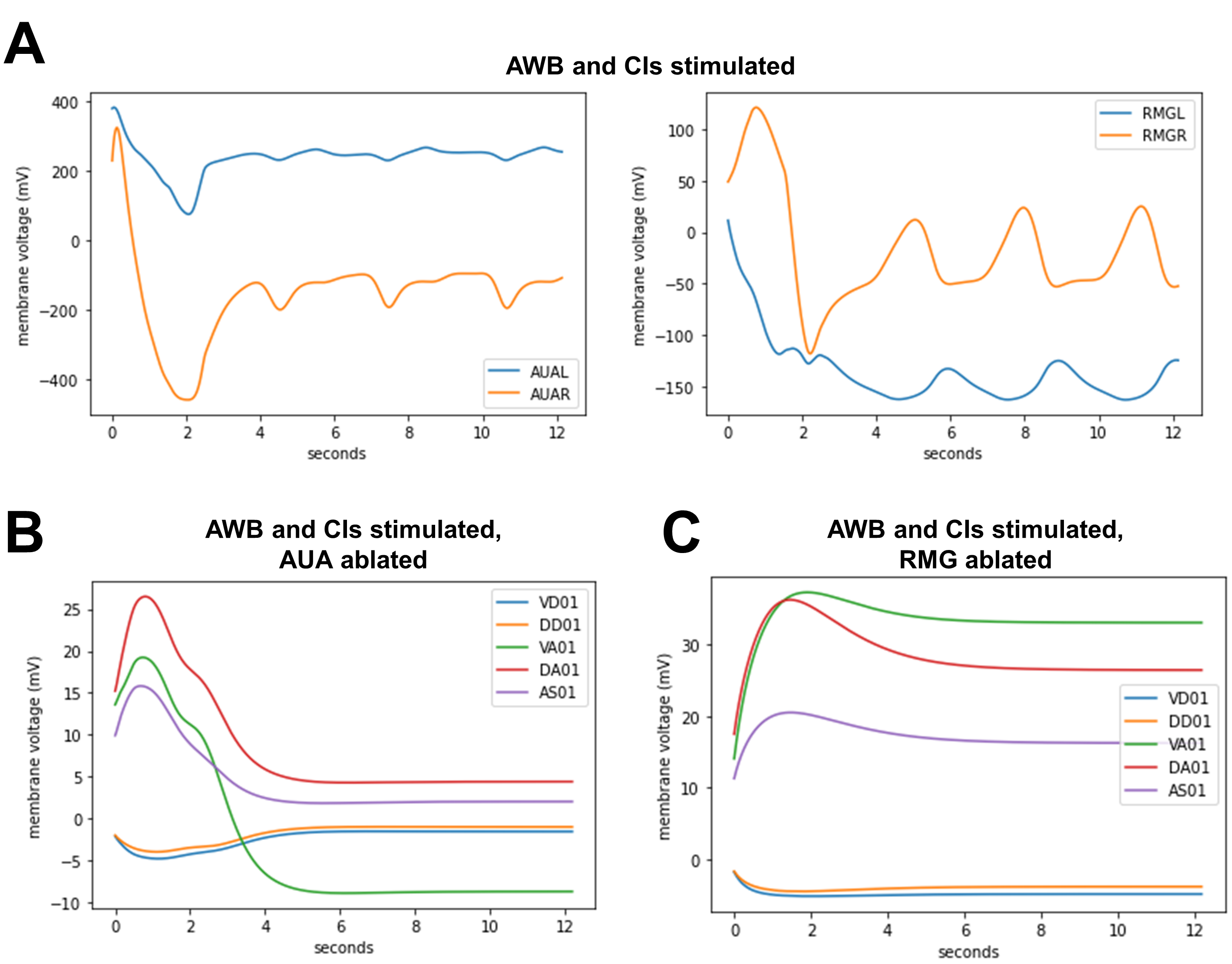

Supplement: Supplementary file 4 — Additional file 4: Fig. S4. AUA and RMG neurons are required for motor neuron oscillations upon AWB and command interneuron stimulation in simulations of the C. elegans nervous system. (A) The same data as in Fig. 3A and B presented as waveforms. (B) The same data as in Fig. 3D presented as waveforms. (C) The same data as in Fig. 3E presented as waveforms. [file 12915_2022_1424_MOESM4_ESM.tif]

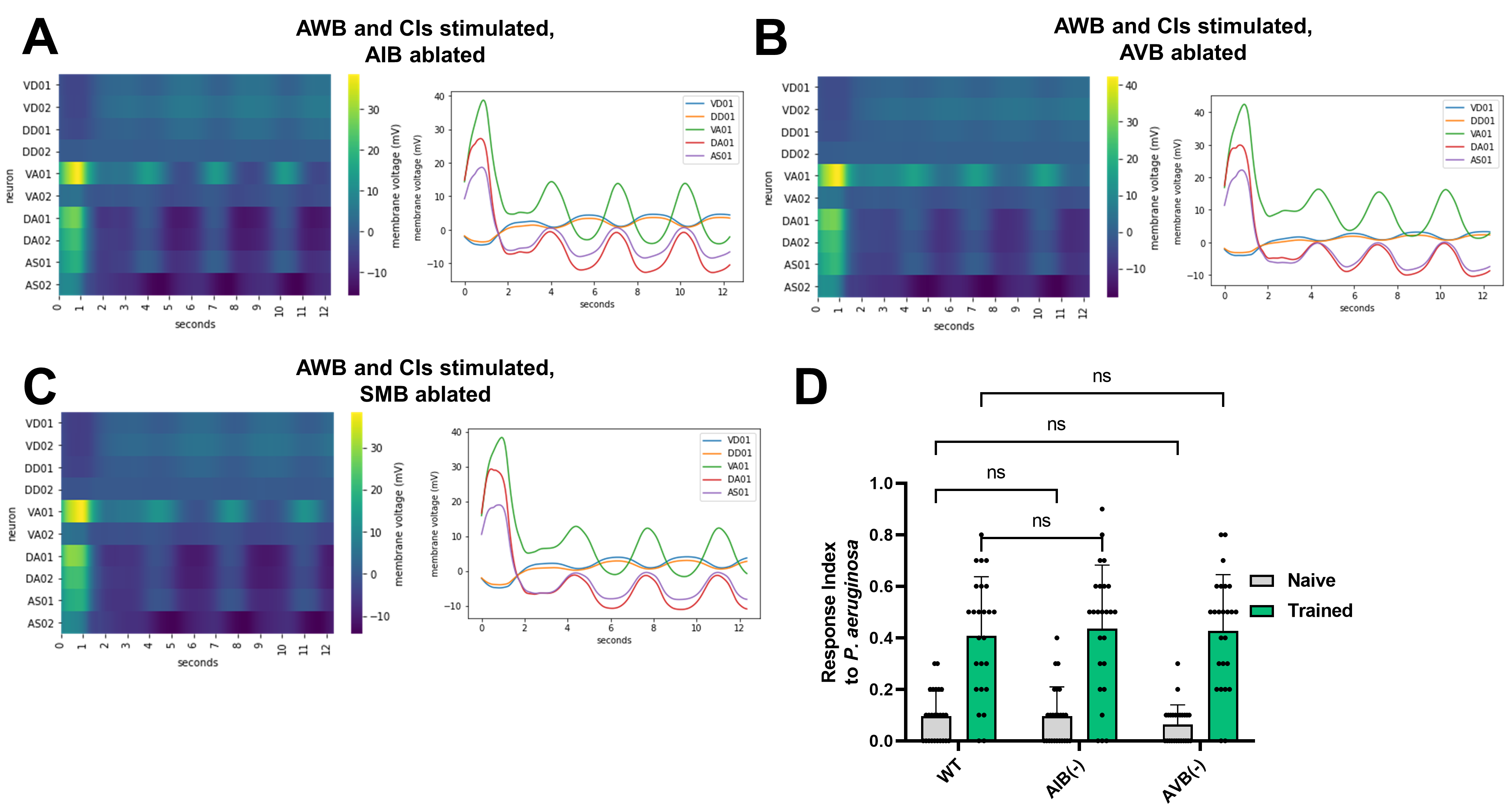

Supplement: Supplementary file 5 — Additional file 5: Fig. S5. AIB, AVB, or SMB neuron ablation does not diminish oscillations in backward locomotion-associated motor neurons. Heatmaps (left) and waveforms (right) of activity of motor neurons (rows) upon 5.0 nA stimulation of AWB neurons and 0.9 nA stimulation of the CIs with AIB (A), AVB (B), or SMB (C) neurons ablated in the Neural Interactome. (D) Response index to P. aeruginosa for both naïve (gray) and trained (green) animals with either no neurons ablated (N2, WT) or AIB (JN578) or AVB (ZM7297) neurons ablated. For ZM7297 animals, the miniSOG ablation protocol was followed as in Fig. 2A. Two-way ANOVA with subsequent comparison to naïve or trained WT groups was performed. Error bars depict standard deviation. N = 25 (individual dots) for all groups. [file 12915_2022_1424_MOESM5_ESM.tif]

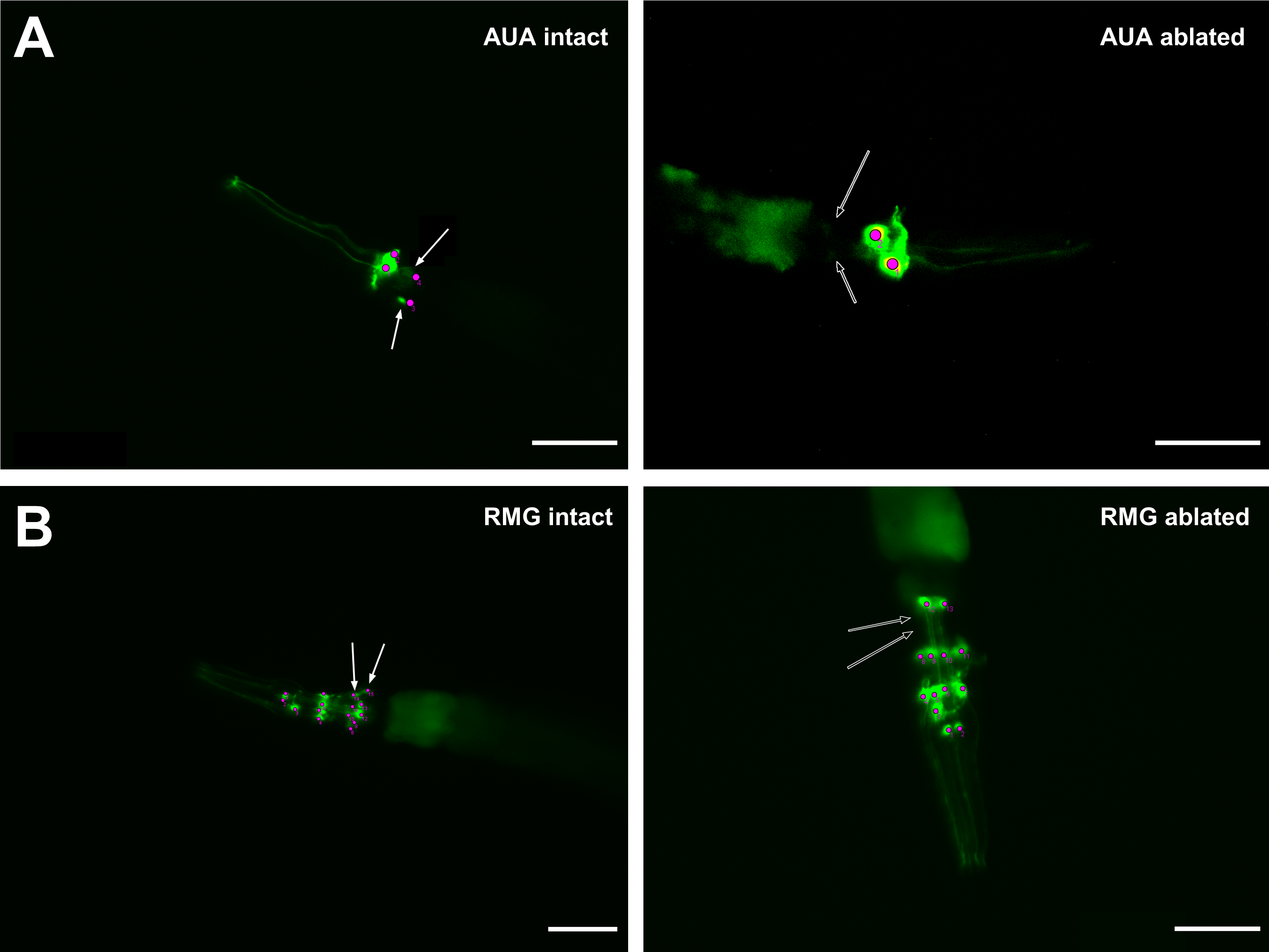

Supplement: Supplementary file 6 — Additional file 6: Fig. S6. Genetic ablation of AUA and RMG neurons. (A) Representative fluorescent micrographs of NY2078 ynIs78 [flp-8p::GFP] (left) and AY178 ynIs78 [flp-8p::GFP]; flp-8p::ced-3 (p15)::nz + flp-32::cz::ced-3 (p17) + unc-122p::rfp (right) animals. (B) Representative fluorescent micrographs of NY2087 ynIs87 [flp-21p::GFP (left) and AY179 ynIs87 [flp-21p::GFP]; flp-21p::ced-3 (p15)::nz + ncs-1p::cz::ced-3 (p17) + unc-122p::rfp (right) animals. White, filled arrows point to intact AUA or RMG neurons, while white, unfilled arrows point to the lack of AUA or RMG neurons. GFP-positive neurons were counted for both intact and ablated animals (pink circles), showing that only the targeted neurons were ablated, with other neurons left intact. AUA intact: 4 GFP neurons; AUA ablated: 2 GFP neurons; RMG intact: 15 GFP neurons; RMG ablated: 13 neurons. Scale bars are 50 μm. [file 12915_2022_1424_MOESM6_ESM.tif]

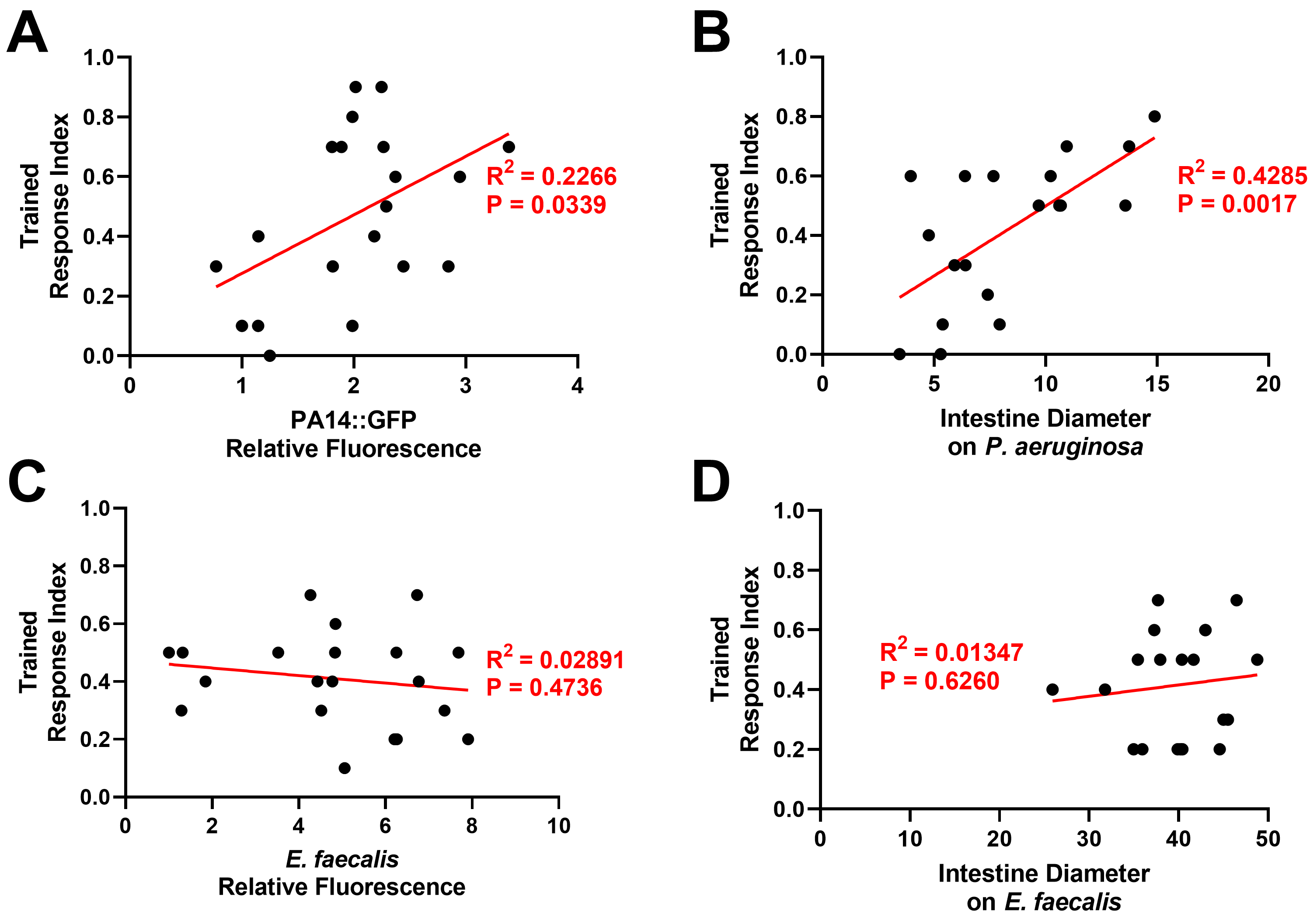

Supplement: Supplementary file 7 — Additional file 7: Fig. S7. Correlation of intestinal distention and learned reflexive aversion for P. aeruginosa but not E. faecalis exposure. (A) PA14::GFP relative fluorescence in the intestine (x-axis) and the trained response index to P. aeruginosa (y-axis) were measured in individual animals (dots), and linear regression was performed (red line). (B) Same as A but with intestinal diameter on P. aeruginosa (x-axis). (C) Same as A but with E. faecalis. (D) Same as B but with E. faecalis. R2 and P-values are shown next to linear regression lines in red. [file 12915_2022_1424_MOESM7_ESM.tif]
